# Physiology and Pathophysiology of Heparan Sulfate in Animal Models: Its Biosynthesis and Degradation

**DOI:** 10.3390/ijms23041963

**Published:** 2022-02-10

**Authors:** Ryuichi Mashima, Torayuki Okuyama, Mari Ohira

**Affiliations:** Department of Clinical Laboratory Medicine, National Center for Child Health and Development, 2-10-1 Okura, Setagaya-ku, Tokyo 157-8535, Japan; okuyama-t@ncchd.go.jp (T.O.); ohira-m@ncchd.go.jp (M.O.)

**Keywords:** heparan sulfate, knockout mice, biosynthesis, lysosome

## Abstract

Heparan sulfate (HS) is a type of glycosaminoglycan that plays a key role in a variety of biological functions in neurology, skeletal development, immunology, and tumor metastasis. Biosynthesis of HS is initiated by a link of xylose to Ser residue of HS proteoglycans, followed by the formation of a linker tetrasaccharide. Then, an extension reaction of HS disaccharide occurs through polymerization of many repetitive units consisting of iduronic acid and *N*-acetylglucosamine. Subsequently, several modification reactions take place to complete the maturation of HS. The sulfation positions of *N*-, 2-*O*-, 6-*O*-, and 3-*O*- are all mediated by specific enzymes that may have multiple isozymes. C5-epimerization is facilitated by the epimerase enzyme that converts glucuronic acid to iduronic acid. Once these enzymatic reactions have been completed, the desulfation reaction further modifies HS. Apart from HS biosynthesis, the degradation of HS is largely mediated by the lysosome, an intracellular organelle with acidic pH. Mucopolysaccharidosis is a genetic disorder characterized by an accumulation of glycosaminoglycans in the body associated with neuronal, skeletal, and visceral disorders. Genetically modified animal models have significantly contributed to the understanding of the in vivo role of these enzymes. Their role and potential link to diseases are also discussed.

## 1. Introduction

**Figure 1:**
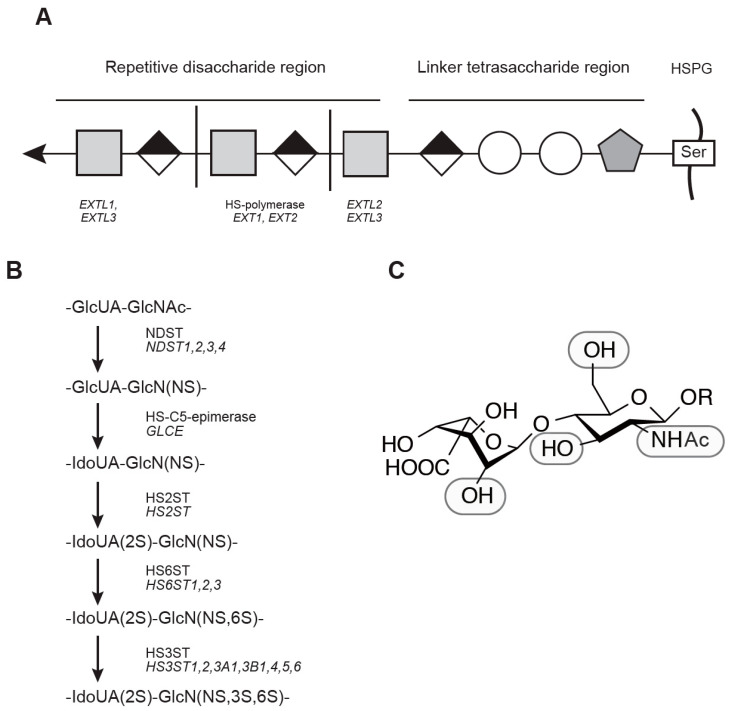
HS biosynthesis. (**A**) Extension of HS from proteoglycan. (**B**) Enzyme reaction of modification in HS. Note that SULF1 and SULF2 are involved in the removal of 6-*O*-sulfate in the last 2 steps. (**C**) Chemical structure of ΔUA-GlcNAc.

**Figure 2:**
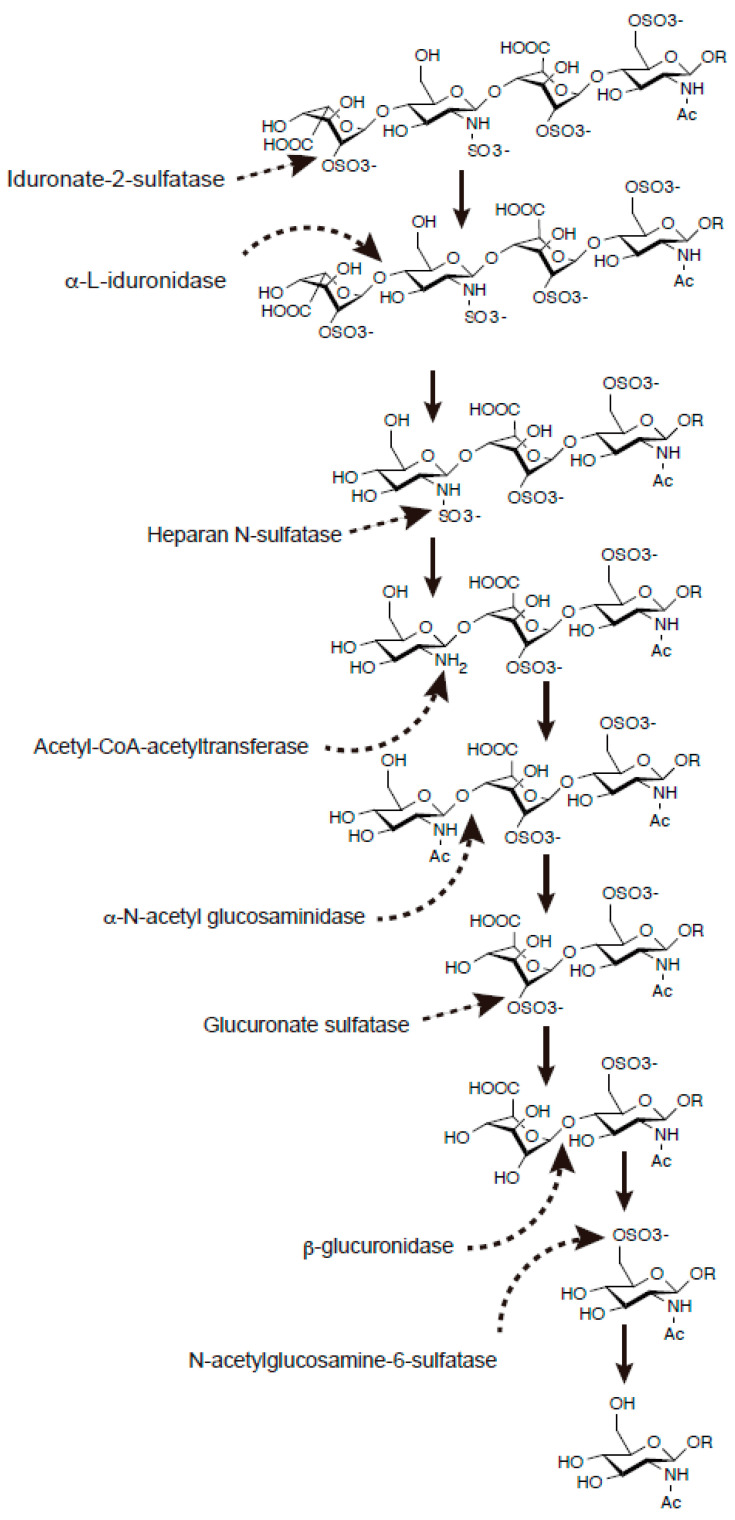
Degradation of HS by lysosomal enzymes involved in mucopolysaccharidosis. Enzymes involved in MPS disease subtypes are as follows: MPS I, α-l-iduronidase; MPS II, iduronate 2-sulfatase; MPS IIIA, heparan N-sulfatase; MPS IIIB, α-*N*-acetylglucosaminidase; MPS IIIC, acetyl CoA: α-glucosaminide acetyltransferase; MPS IIID, *N*-acetylglucosamine 6-sulfatase; MPS VII, β-glucuronidase.

Heparan sulfate (HS) is a type of glycosaminoglycan (GAG) that contains many *O*-(1→4)-linked uronic acid and a glucosamine [1,2]. HS is widely found in tissues, playing an essential role in maintaining cellular function. In particular, the central nerve system (CNS), bone, immune system, and tumor metastasis have functional relevance to HS under (patho)physiological conditions. HS-proteoglycans (HSPGs) are proteins that are *O*-linked to HS through Ser residue in the core protein. The biochemical property of proteoglycan was extensively studied about four decades ago by Yanagishita and Hascall [3,4]. In contrast, HS-binding proteins are rather ambiguously defined. One clearly defined example includes a definition of any protein that binds to Heparan-Sepharose and dissociates from the resin by an increasing concentration of salt under neutral pH [2]. Thus, the biological effect of HS is exerted by modulation of the interaction between HSPG and HS-binding protein. The core protein of HSPG is biosynthesized in the ribosome followed by translocation to the endoplasmic reticulum (ER) and Golgi, where *O*-linked attachment of xylose followed by extension of HS takes place (Figure 1A). Following the formation of linker tetrasaccharide and an extension of glucuronic acid (GlcA) and *N*-acetylglucosamine (GlcNAc), the modification of HS by sulfation, epimerization, and desulfation modifies the bioactivity of HS (Figure 1B). In particular, there are specific enzymes that sulfate at *N*-, 6-*O*, and 3-*O* of GlcNAc and 2-*O* of iduronic acid (IdoA) (Figure 1C). Apart from HS biosynthesis, it may be aberrantly accumulated under pathophysiological conditions. A well-known example includes mucopolysaccharidosis (MPSs), a group of genetic disorders that fail to properly degrade HS in the lysosome (Figure 2). While these disorders are rare, effective treatments have been developed. Enzyme replacement therapy infuses a therapeutic enzyme agent intravenously. A notable phenotype involves CNS involvement, visceral manifestations, and skeletal deformation. Among these three manifestations, enzyme replacement therapy effectively improves visceral manifestation, contributing to improving the quality of life of affected individuals.

From a biochemical point of view, the position and number of *N*- and *O*-sulfate are often critical to understand biochemical property of HS. For this purpose, heparitinases with altered substrate specificity are generally used in combination. For example, heparitinase I reacts with a relatively short GAGs, whereas heparitinase III favors a larger GAG substrate. Due to the absence of distinct chromophore in HS, there are many techniques to detect HS in biological samples. Classically, fluorometric derivatization has often been used. Recently, mass spectrometric detection has become an increasingly common technique for the quantification of low molecular weight compounds. This technique also allows us to quantify HS disaccharide species in their intact or derivatized form. The intact HS disaccharides are normally chromatographed using graphite carbon-based separation [5]. In order to obtain higher sensitivity, these compounds may be derivatized using a variety of reagents, such as 3-methyl-1-phenyl-5-pyrazolone [6,7]. In this reaction, the best result is obtained under alkali conditions [8]. Alternatively, methanolysis may be used for quantification of HS disaccharide [9,10]. In this case, the COOH moiety of uronic acid and one glycosylation bond were methylated during the reaction. Such a technique has been used for the diagnosis of MPS-affected individuals.

Heparin is an HS-related biopolymer with different biochemical properties. First, heparin has a much smaller molecular weight (i.e., 10–15 kDa) compared with that of HS (15–25 kDa). The average number of sulfate moiety in disaccharide in heparin is two to three compared with one to two in HS. Heparin is biosynthesized in connective tissue-typed mast cells while HS is formed in almost all cells. Heparin has a strong anticoagulant action through the activation of antithrombin. For commercial use, porcine intestine mucosa has been used as the source of heparin.

## 2. Biosynthesis of HS and Phenotype of Mice Deficient in HS Biosynthesis Enzyme Genes

HS is *O*-linked through Ser residue to core protein, HSPG (Figure 1). This reaction occurs in the ER and Golgi apparatus. Matured HSPG localizes either to the membrane or extracellular space. HS degradation occurs in the lysosome, a cellular compartment with an acidic environment (Figure 2). HS has many biological properties, including organ formation, the regulation of signal transduction, and the invasion and translocation of tumor. It is also known that some bacteria and viruses use HS as their receptors. For example, HS enhances corona virus infection [11]. Thus, apparently, most of the biological effects of HS may be exerted by functional modulation between HSPG and HS-binding protein. HS species with no sulfation are prominent in dried blood spots, and this is also applicable to many tissues [12,13].

### 2.1. Linker Tetrasaccharide Formation and Disaccharide Extension

Heparan sulfate has a unique four oligosaccharides GlcA-Gal-Gal-Xyl, of which the reducing end Xyl is linked to Ser residue of proteoglycans (Figure 1A) [14]. A GlcNAc is then attached to this end for HS biosynthesis. Subsequently, the GlcA and GlcNAc are alternatively extended by GlcA transferase and GlcNAc transferase, respectively. A complex of EXT1/EXT2 enzymes catalyzes both GlcA/GlcNAc transferase reactions. EXTL2 has a GlcNAc transferase activity that terminates HS chain polymerization by the addition of GlcNAc at the linker tetrasaccharide that has been Xyl-phosphorylated by FAM20B [15]. In contrast, EXTL3 also has a GlcNAc transferase activity that similarly targets linker tetrasaccharide without Xyl phosphorylation [16]. As a result, subsequent HS polymerization by EXT1/EXT2 enzymes continues. While no study has reported the phenotype of EXTL1, B cell-specific expression of EXTL1 in mice showed a partially impaired B cell maturation [17].

A genetic defect in these genes in mice revealed that these mice are lethal [18] (Table 1). Another phenotype apparently involves abnormal chondrogenesis [19,20]. This was supported by a lot of evidence of a variety of conditional knockout mice. Essentially, a defect in linker tetrasaccharide formation results in osteochondroma or related chondrocyte hypertrophy. This phenotype has been extensively studied because a similar phenotype was found in humans [21]. The Ext1-dependent heparan sulfate regulates the range of IHH signaling during endochondral ossification [22]. The defect in HS formation was also affected by the BMP-mediated signaling pathway [23]. While physiological levels of HS disaccharide in chondrocytes were approximately 1% of that of CS disaccharide under normal conditions, total removal of HS through a deficiency of linker tetrasaccharide by the genetic technique caused a severe defect in chondrogenesis [24]. Other than that, abnormal neurogenesis and immune modulation have been reported.

### 2.2. Modification Reactions

#### 2.2.1. *N*- and *O*-sulfation

HS is susceptible to *N*- and *O*-sulfation. There are four *N*-deacetylase/*N*-sulfotransferases (NDSTs), one 2-*O*-sulfotransfease (HS2ST), three 6-*O*-sulfotransferases (HS6ST), and seven 3-*O*-sulfotransferases (HS3ST) in humans and mice (Figure 1B). Because these enzyme reactions are not strictly sequential, there is variation of the HS disaccharide in terms of the position and degree of sulfation. NDST plays a key role in immune modulation. In contrast, both HS2ST and HS6ST affect the tubular formation of mesenchymal organs.

##### NDST

Similar to a mouse lacking EXT1/EXT2 enzymes, the lack of the NDST1 enzyme leads to neonatal lethality [53]. These mice also have a defect in bone and CNS development [55]. The defect in NDST enzyme leads to an increasing accumulation of heparin in mast cells, suggesting that *N*-sulfation could activate downstream sulfatases, such as HS2ST, HS6ST, and HS3ST, by an uncharacterized mechanism [134].

Apart from these globally *Ndst1*-deficient mice, the distinct role of NDST is best described in immune modulation. This is correlated with the expression of a chemokine receptor on endothelial cells, where its contact with leukocytes plays a key role in leukocyte migration. Notably, a recent report described that a conditional NDST1-deficient mouse exhibited impaired rejection in an acute renal allograft model [72].

##### HS2ST

HS2ST is a unique sulfotransferase that specifically catalyzes 2-*O*-sulfation in mammals (Figure 1C). In *Hs2st(−/−)* mice, 2-*O*-sulfate HS disaccharide was absent while compensatory accumulation of *N*- and 6-*O*-sulfate was reported [135]. These mice are embryonic lethal with renal agenesis [102]. There are several defects in CNS, such as retinal axon guidance [104], astroglial translocation [108], and facial branchiomotor neurons in the hindbrain [107]. A study using conditional knockout mice revealed that both the endothelial expression of HS2ST attenuates the rolling velocity of neutrophil and enhances IL-8- and MIP-2-induced neutrophilic infiltration [73]. Consistent with this immunomodulatory role, reduced formation of the neutrophil extracellular trap with increasing binding to group B Streptococcus was reported [109]. Thus, HS2ST also plays an important role in immunity to infections. Attenuated expression of *Hs2st* in gene trap mice was shown to impair the development of the kidney [102] and laminal grands [110]. No chondrocyte phenotype was reported in *Hs2st(−/−)* mice.

##### HS6ST

HS6ST introduces a sulfate at the 6-*O* position of *N*-acetylgluctosamine in HS. This position has been suggested as a critical point for the interaction with FGF2 receptors that ultimately leads to activation of Erk [136]. In *Hs6st1(−/−)* mice, abnormal axon patterning in retinal ganglion cells [104], cranial axon guidance [107], and corpus callosum development [105] are the reported CNS phenotypes in murine models. Although there was no detailed study for these phenotypes, a lack of HS6ST1 enzyme might modulate the FGF2-mediated signaling pathway in neural cells, such as neuronal stem/progenitor cells. In *Hs6st1(−/−);Hs6st2(−/−)* double knockout mice, the percentage of 6-*O*-sulfation was almost completely abolished, with reduced tryptase activity in fetal skin-derived mast cells [114]. Because mast cells are heparin-producing cells [91], as described, thus attenuated production of heparin in these mice could either inhibit the gene expression or the extracellular release of trypase in this model.

##### HS3ST

HS3ST has been suggested to be associated with the generation of heparin because heparin is heavily sulfated compared with heparan sulfate. Specifically, the 3-*O* position of galactose is sulfated in heparin by enzymatic action of HS3ST. The *Hs3st1(−/−)* mice were established [116]. They exhibited a reduced antithrombin-binding area in the carotid artery with normal tissue fibrin accumulation. In an LPS-challenged model, an increasing sensitivity to TNF-α was reported, suggesting an enhanced immune reaction in these mice [115]. In *Drosophila*, a reduction of *Hs3st-B*, an ortholog of *Hs3st3b1* in humans, leads to a neurogenic phenotype through Notch signaling [137]. However, a similar phenotype in humans was not reported.

#### 2.2.2. Glucuronic Acid C5-epimerization

Glucuronic acid C5-epimerase (GLCE) is a unique enzyme that catalyzes the epimerization of glucuronic acid to iduronic acid [138]. This conversion occurs by catalyzing the isomerization of an equatorial COOH in glucuronic acid at C5 to an axial COOH in iduronic acid. The resulting sulfate group at the C2-position of iduronic acid prevents C5-epimerization from catalyzing reverse epimerization [139]. Apart from the biochemical reaction that occurs in animals and in vitro assays, where a relatively small quantity is sufficient, maximization of the biochemical product is an important issue from an industrial point of view. This is also applicable to the production of heparin, an anticoagulant used clinically. Apparently, a lesser content of C2-sulfate in iduronate or higher enzyme activity of GLCE yields greater heparin production on a biochemical basis.

GLCE(−/−) mice are neonatally lethal due to respiratory failure [95]. In these mice, kidney formation was not observed; delayed development of the lung and bone has also been reported. Apart from these in vivo observations, a biochemical examination revealed a lack of iduronic acid in these mice. For heparin biogenesis, GLCE is unambiguously essential [99]. In the most recent study, histological analysis of the lungs in embryos revealed no difference in the morphology between wild-type and mutant animals up to E16.5 [101]. However, the distal lung of E17.5-18.5 mutants is still populated by epithelial tubules, lacking the typical saccular structural characteristic of a normal E17.5 lung. Further immunostaining revealed strong signals of surfactant protein-C but a weaker signal of T1α/podoplanin in the mutant lungs in comparison with wild-type littermates, suggesting that the differentiation of type I alveolar epithelial cells was impaired. As a potential mechanism, it has been discussed that the reduced vascularization in the developing lungs could be associated with a failure of maturation of these cells.

#### 2.2.3. Sulfatase Reaction

In humans and mice, there are two sulfatases that catalyze the sulfatase reaction at the 6-*O* position of GlcNAc. This reaction occurs only after sulfation reactions are complete. SULF1 and SULF2 are enzymes catalyzing the removal of 6-*O* sulfate of mature HS. These enzymes are located extracellularly, and their function has been postulated as being the regulators of cellular function by maintaining the appropriate amount of sulfate. These enzymes are active under neutral pH; thus, they are distinct from lysosomal sulfatase.

*Sulf1(−/−)* and *Sulf2(−/−)* have defects in CNS development. In *Sulf1(−/−)* mice, the neural progenitor cells to motor neuron progenitor are increasing while those to oligodendrocyte progenitor are decreasing [118,120]. Furthermore, there are two additional CNS phenotypes. One report describes impaired axon guidance in the corticospinal tract [128]. A more recent study demonstrated impaired motor functions in these mice [129], with *Sulf1(−/−);Sulf2(−/−)* double-deficient mice exhibiting the most severe phenotype involving embryonic development that is similarly found in *Ext1/Ext2-* and *Ndst*-deficient mice. Furthermore, the chondrogenic phenotype was also reported in *Sulf1(−/−);Sulf2(−/−)* double-deficient mice. Interestingly, in a gene trap (gt) experiment that inactivates *Sulf1* and *Sulf2* (i.e., *Sulf1(gt/gt)* and *Sulf2(gt/gt)*), these mice were lethal with abnormal chondrocyte development, suggesting that a small residual enzyme activity of sulfatase enzyme is essential for embryonic lethality in mice [124]. Lastly, it is often observed that the phenotype of disease models may be altered depending on the genetic background. This is also the case in these sulfatases; namely, *Sulf1(−/−);Sulf2(−/−)* double-deficient mice on a C57BL/6 genetic background are lethal while those on a C57BL/6 and ICR mixed genetic background are not [129,140].

### 2.3. Diseases in Humans

Disorders identified in the genes for HS biotransformation are rare in both HS-biosynthesis and HS degradation. Exostosis is a rare disease that is caused by dominant mutation of EXT1/EXT2 enzymes in humans [21,141]. Disorders caused by *N*- and *O*-sulfotransferases, C5-epimerase, and 6-*O*-sulfatases have not been described in humans.

## 3. Lysosomal Storage Disorders (LSDs)

A lysosome is a cellular compartment that hydrolyzes multiple biomolecules, such as oligosaccharides, lipids, glycolipids, sphingolipids, and mucopolysaccharides [142,143]. It is well known that the pH of a lysosome is maintained in the acid range. To achieve this, lysosomal v-ATPase is an essential molecular machinery that incorporates H^+^ inside the lysosome at the expense of adenosine triphosphate. The failure in the proper regulation of this hydrolyzing activity leads to an accumulation of biomolecules in the lysosomal vacuoles. Lysosomal degradation is widely found in macrophages and phagocytes. For the proper lysosomal targeting of enzymes and proteins, these biomolecules are normally post-transcriptionally modified with mannose-6-phosphate. This ligand, mannose-6-phosphate, binds to a cation-independent mannose phosphate receptor that is expressed on the lysosomal membrane. Thus, once a mature LSD enzyme is endocytosed, this enzyme is selectively targeted to the lysosome. This mechanism is known as cross-correction [144]. Today, a lot of therapeutic strategies, such as hematopoietic stem cell transplantation, enzyme replacement therapy, and gene therapy, have been developed based on this mechanism.

LSDs are characterized by a deficiency of lysosomal enzymes, associated with an accumulation of sphingolipids, glycolipids, glycosaminoglycans, and other biological compounds [145]. We now know that approximately 50–60 genes are involved in this disorder [143]. The prevalence of classical LSDs is normally very rare, but it increases significantly when the population contains a high rate of late-onset disorders. LSDs exhibit a variety of manifestations involving the CNS, bone, and hepatosplenomegaly, but CNS involvement is commonly observed. Although the gene responsible for each disease has been identified, the cause of the disorder is not well understood. As a result of genetic surveys, accumulated evidence has indicated that there are many small populations that have a unique pathogenic mutation for LSDs. This is most evident in populations with high rates of consanguineous marriages. Among various mutations, missense mutation is commonly found in affected individuals, but this generally exhibits a milder phenotype. In contrast, a severe phenotype is usually associated with gross deletion, frameshift, recombination, and other mutations. Nonsense mutation usually leads to a severe phenotype, but occasionally it shows a milder phenotype. This partly occurs based on a mechanism called “read-through”, where tRNA recruits an irrelevant amino acid as expected from the triplet codon. For example, the IDS R8X (c.22C>T) mutation is such a missense mutation where a termination codon appears immediately after the translation start site [146,147]. Some patients survived at the time of the survey without cognitive decline, a representative clinical manifestation of the mild type of MPS II [146]. The frequency of read-through is low in all missense mutations but is occasionally identified by genetic testing. The reason why this is not a general mechanism remains largely unknown. To facilitate read-through, some antibiotic agents, such as aminoglycoside, are used in vitro [148].

Mucopolysaccharidosis (MPS) is characterized an accumulation of glycosaminoglycans in the body that affects multiple organs (Figure 2). There is a distinct substrate specificity of enzymes responsible for pathogenesis. MPS I and II are caused by α-l-iduronidase and iduronate-2-sulfatase that accumulate dermatan sulfate (DS) and HS at the same time. Four disease subtypes of MPS III, namely MPS IIIA-D, are caused by *N*-sulfoglucosamine sulfohydrolase, α-*N*-acetylglucosaminidase, heparan-α-glucosaminide *N*-acetyltransferase, and *N*-acetylglucosamine 6-sulfatase, respectively, which specifically increase HS. MPS IVA is caused by *N*-acetylgalactosamine-6-sulfatase, which increases keratan sulfate. MPS VI is caused by *N*-acetylgalactosamine 4-sulfatase, which increases DS. MPS VII is caused by β-glucuronidase, which increases DS, HS, and hyarulonate.

A widely accepted therapy for LSDs includes enzyme replacement therapy, which infuses recombinant human LSD enzyme [143]. Its efficacy has been demonstrated in Pompe, Fabry, and Gaucher disease and MPS I, II, IVA, and VI, respectively [142]. Among various manifestations, visceral disorder, such as hepatosplenomegaly, is a good target for this therapy. Accumulating earlier studies have demonstrated a younger brother or sister exhibits better treatment results, especially when the older brother or sister has been diagnosed. Based on this evidence, it is known that the pharmacological outcome for LSDs may be maximized when the treatment begins during an asymptomatic period [149]. Consistently, newborn screening, a public health program to identify an affected newborn in the population, has been implemented in the US, Taiwan, and other countries [150,151,152]. This outcome of newborn screening is generally satisfactory, especially in the US, because prompt treatment becomes available. For more than a decade, an intravenously administered therapeutic enzyme has been the only bio-engineered strategy for treatment, including CNS. Based on recent studies about the receptors on pericytes, a recombinant enzyme, fused to anti-human monoclonal antibody against these endogenous receptors has been examined with satisfactory results [153].

Importantly, lysosomal biogenesis occurs before an LSD protein becomes pathogenic [154]. Transcription factor EB (TFEB), one of three similar transcription factors in humans and mice, is an essential transcription factor that is closely associated with lysosomal biogenesis. In a resting state, TFEB is serine-phosphorylated at multiple sites by mTORC1. Among them, Ser211 has been considered the key phosphorylation site that is bound to an adaptor protein 14-3-3 [155]. When TFEB is dephosphorylated by a phosphatase calcineurin, cytosolic TFEB translocates into the nucleus, followed by an initiation of gene expression involved in both lysosomal enzymes and biogenesis. For example, the expression of LAMP2, a lysosomal membrane protein often used as a biomarker, is regulated by the TFEB-induced gene expression mechanism. Similarly, β-hexosaminidase is an LSD-related gene responsible for Sandhoff disease, another well-known biomarker for LSD. In this case, the level of GM2, a ganglioside generated from hexosaminidase enzyme reaction, is elevated as well.

## 4. MPS Type II

### 4.1. Pathophysiology

MPS II (OMIM 309900) is an X-linked disorder characterized by a deficiency in enzyme activity of iduronate-2-sulfatase (IDS, EC 3.1.6.13, Figure 2) [156]. IDS catalyzes the elimination of sulfate at the 2-*O* position of iduronic acid from GAGs, such as dermatan sulfate and heparan sulfate. The major manifestations include hepatosplenomegaly, skeletal deformities, valvular heart disease, enlarged tongue, upper airway obstruction, and abnormal dentition. The phenotype of MPS II is linked to an accumulation of GAGs in tissues. Importantly, approximately 70% of individuals have CNS involvement. In the case of the *IDS* gene, there is a pseudogene for the *IDS*, termed the *IDS2*, which lacks enzyme activity [146]. Although many missense mutations have been identified, it has frequently been found that the *IDS* mutations involve recombination, a gross deletion, and a frameshift. This is well correlated with the fact that CNS involvement is the major manifestation in this disorder. The tertiary structure of the catalytic center has long been postulated based on the similarity of the amino acid sequence and a reported tertiary structure of aryl sulfatase. Recently, crystallographic data for the IDS enzyme became available [157]. Based on this result, most missense mutations have been found in the cytosolic, transmembrane, or interface regions of the IDS protein. Mammalian sulfatases, such as IDS, arylsulfatase A and B, and cholesterol sulfatase, are known to be involved in the post-transcriptional modification of cysteine residue at the catalytic center. Formyl glycine is a modified amino acid essential for the catalytic activity of sulfatases. For this reaction, sulfatase-modifying factor 1 plays a key role [158]. In fact, the loss of this enzyme’s activity leads to multiple sulfatase deficiencies, another LSD. In vitro, the co-expression of sulfatase and sulfatase-modifying factor 1 enhances sulfatase activity, raising the possibility that sulfatase agents might require full enzyme activation for therapeutic application.

### 4.2. Phenotype of Mouse Model

A mouse model lacking IDS activity has been established [159]. Although several lines have been reported to date, the best studied mouse model contains an allele of which exons 4 and 5 have been replaced with a neomycin resistance gene. The major phenotype includes splenomegaly, skeletal deformities, microgliosis, astrocytosis, and abnormal CNS function. The efficacy of many therapies, such as bone marrow transplantation, enzyme replacement therapy, and gene therapy, has been tested in this model. Among these manifestations, skeletal deformity is linked to an elevation in DS [159]. In contrast, CNS involvement is linked to an elevation in HS. When the efficacy of a recombinant enzyme of IDS fused to an antibody against human transferrin receptor was tested in a murine model, a novel IDS model expressing human transferrin by a knock-in strategy was used [153].

### 4.3. Treatment

The current standard therapy for MPS II is an enzyme replacement therapy that infuses a therapeutic recombinant enzyme intravenously [160]. This treatment has had a positive therapeutic outcome in somatic, but not neurological, manifestations. The latter is due to the blood–brain barrier, which separates the blood and brain with neuronal endothelial cells. To overcome this difficulty, the possibility of intrathecal administration has been examined [161].

### 4.4. Biomarker

An accumulation of GAGs is a hallmark of MPS II and other disease subtypes of MPS ranging from MPS I to MPS VII. Historically, an elevation in the colorimetric substance in the presence of Alcian blue or Methylene blue has been used as a measure of GAG accumulation in clinical specimens. This is based on the ionic interaction between the sulfate group and positively charged nitrogen atoms in these dyes under neutral pH. For classification, electrophoresis has been used for the separation of DS, HS, CS, and keratan sulfate. In MPS II, together with MPS I, both HS and DS are elevated under pathophysiological conditions. In contrast, HS, DS, and keratan sulfate alone are preferentially elevated in MPS III, MPS VI, and MPS IV, respectively. These biomarkers may be quantified as a disaccharide after digestion with acid or enzyme reaction. Methanolysis and other alcoholysis of GAG into disaccharides may be performed more affordably, but quantification requires controlled experimentation because formed disaccharides undergo further degradation after a prolonged reaction period [9]. Enzyme digestion of GAG occurs under much milder conditions, such as neutral pH, but the enzymes used for this digestion have limited availability [162].

## 5. Future Perspectives

These animal models are anticipated to be applied for the development of novel therapeutic agents. As mentioned, enzyme replacement therapy has been developed using these mouse models. Gene therapy that delivers therapeutic genes exogenously has attracted a lot of attention in LSD and other clinical areas. Historically, the use of lentiviral vector has been studied extensively. This technique uses autologous hematopoietic stem cells for delivery; thus, affected individuals do not need to wait long to find an HLA type-matched donor and have a reduced risk for graft-versus-host disease. More recently, adeno-associated virus (AAV) has also attracted attention for gene therapy because these vectors are not incorporated into genomes, thus the descendants of affected individuals do not have a therapeutic gene [163]. Among more than 10 serotypes of AAVs, serotype 9 has often been used because its penetration of the blood–brain barrier is better than the others.

Similarly, the roles of disease modifiers will be examined in the future. Among the reported phenotypes, CNS involvement in LSDs has suggested an association with inflammation because microglial activation is commonly found in mice and humans. A recent study in macrophage biology has suggested that macrophages can be grouped into two distinct subpopulations, such as inflammatory M1 macrophages and anti-inflammatory M2 macrophages [164]. In this classification, M1 macrophages produce inflammatory cytokines, such as TNF-α, IFN-γ, IL-6, and others, while M2 macrophages produce IL-10 and TGF-β and express mannose receptors and scavenger receptors. Thus, a systematic study of neuroinflammation in CNS involvement in LSD is required. Apart from inflammation, X-inactivation is involved in the disease initiation in females with X-linked disorders, such as MPS II in LSDs. In fact, such an example has been reported in humans, although the prevalence is low. For diagnosis, the severity of the manifestation in X-linked disorders in females is commonly dissociated with the changes in biomarkers, such as accumulating substances and enzyme activity. In future studies, the molecular mechanism behind female MPS II needs to be elucidated.

## Figures and Tables

**Table 1:** Phenotype of mice that lack HS biosynthesis enzymes.

	Mouse Phenotype				Description	Reference
	Lethality	Abnormal Chondrogenesis	Neural Disorders	Immunomodulation		
Ext1, Ext2, Ext2l, Ext3l						
*Ext1(−/−)*	Let				Embryonic lethal	[18]
*Ext1(+/−)*		chondr			↑Exostosis-like phenotype	[25]
*Ext1(+/−)*					↑Ihh signaling; ↑chondrocyte proliferation; ↓hypertrophic differentiation	[26]
*Ext1(+/−);Sgsh(−/−)*					Normal MPS IIIA pathogenesis	[27]
*Ext1(gt/gt)*	Let	chondr			Embryonic lethal	[22]
*Ext1(gt/gt)*					↑Chondroitin sulfate	[24]
*Ext1(gt/gt)*					↑BMP signaling in engineered cartilage	[28]
*Col2-Cre;Ext1(f/f)*		chondr			↓Bone growth; ↓chondrocyte hypertrophy	[29]
*Col2-Cre;Ext1(f/f)*		chondr			↑Multiple osteochondromas	[30]
*Col2-Cre;Ext1(f/+)*		chondr			↑Osteochondromas	[25]
*Col2a1-Cre;Ext1(f/f)*		chondr			↑Osteochondromas; ↑phosphorylation of Smad1/5/8	[31]
*Dermo1-Cre;Ext1(f/+)*		chondr			↑Osteochondromas	[25]
*Fsp1-Cre;Ext1(f/f)*		chondr			↑Osteochondromas	[32]
*Gdf5-Cre;Ext1(f/f)*		chondr			↓Proximal limb joints	[33]
*Prg4-Cre;Ext1(f/f)*		chondr			↑Hypertrophic chondrocyte; ↑cartilage thickness	[34]
*CaMKII-Cre2834;Ext1(f/f)*			Neural		↓Excitatory synaptic transmission	[35]
*Wnt1-Cre;Ext1(f/f)*			Neural		↓Commissural axon path finding	[36]
*Lck-Cre;Ext1(f/f)*				Immuno	↑DN4 cells in thymocytes	[37]
*Pdgfrα-Cre;Ext1(f/f)*				Immuno	↓Size of fetal thymus organ cultures	[38]
*Shh-Cre;Ext1(f/f)*					↓Cell proliferation in stomach; ↓FGF signaling	[39]
*Shh-Cre;Ext1(f/f)*					↓Shh signaling; ↑branching tips; ↑branching number	[40]
*2.5P-Cre;Ext1(f/f)*					Abnormal podocyte morphology; → albuminuria	[41]
*Col2-rtTA-Cre;Ext1(e2neofl/e2neofl)*	Let	chondr			↑Multiple osteochondromas	[19]
*Col2-rtTA-Cre;Ext1(e2fl/e2fl)*				Immuno	↓Osteoarthritis	[42]
*Tek-rtTA+;Tet-Cre;Ext1(f/f)*				Immuno	↓Chemokine presentation in epithelial cells; ↓lymphocyte homing	[43]
*Krt14-rtTA;Tet-Cre;RosaLSL;Ext1(f/f)*					↓Corneal epithelium; ↓epithelial layers	[44]
*Ext2(−/−)*		chondr			↑Exostoses	[20]
*Ext2(−/−)*					↓FGF signaling in mutant embryo	[45]
*Ext1(+/−);Ext2(+/−)*		chondr			↑Exostosis-like phenotype; ↓heparan sulfate	[25]
*Ext1(+/−);Ext2(+/−)*					↓Sodium storage capacity in skin; ↓endothelial surface layer thickness	[46]
*Ext1(+/−);Ext2(+/−);Sgsh(−/−)*			Neural		↓MPS IIIA pathogenesis	[27]
*Extl2(−/−)*			Neural		↑Chondroitin sulfate proteoglycans deposition	[47]
*Extl2(−/−)*				Immuno	↑Incidences in non-alcoholic steatohepatitis and hepatocarcinoma	[48]
*Extl2(−/−)*				Immuno	↓Body weight; ↓hepatocyte in CCl4-induced liver failure	[49]
*Extl2(−/−)*					↑Aortic calcification in chronic kidney disease	[50]
*Extl3(−/−)*	Let				embryonic lethality	[51]
*Nphs1-Cre;Extl3(f/f)*					→Urinary albumin excretion	[52]
*N*-Deacetylase/*N*-sulfotransferases 1–4						
*Ndst1(−/−)*	Let				↑Respiratory distress syndrome	[53]
*Ndst1(−/−)*		chondr			↓Defective skull development	[54]
*Ndst1(−/−)*		chondr	Neural		↓Cerebral development; ↓craniofacial development	[55]
*Ndst1(−/−)*				Immuno	↓Binding affinity to vaccinia virus; ↓binding affinity to myxoma virus.	[56]
*Ndst1(−/−)*					↑Pericyte detachment; ↓pericyte migration	[57]
*Ndst1(−/−)*					↓Ca^2+^ kinetics in myotubes	[58]
*Ndst1(−/−)*					↓Differentiation of lung cells; ↑cell proliferation.	[59]
*Ndst1(−/−)*					↑Glomerular hypertrophy in the kidney; ↓podocyte organization	[60]
*Ndst1(−/−)*					↓Podocyte-matrix interaction	[61]
*Ndst1(−/−)*					↓Lens development	[62]
*Ndst1(−/−)*					↓Heart development	[63]
*Ndst1(−/−)*					↓ERK signaling in lacrimal gland bud	[64]
*Ndst1(+/−)*		chondr			↓Osteoarthritis	[42]
*Col2-Cre;Ndst1(f/f)*		chondr			↓Osteoarthritis	[42]
*L7-Cre;Ndst1(f/f)*			Neural		→Purkinje cell development	[65]
*Olig2-Cre;Ndst1(f/f)*			Neural		↑Lesion size; ↑reactivity of microglia and oligodendrocyte precursor cells in myelin destruction	[66]
*CD11c-Cre;Ndst1(f/f)*				Immuno	↓Lewis lung carcinoma growth; ↑tumor-associated CD8+ T cells	[67]
*Tek-Cre;Ndst1(f/f)*				Immuno	↓Leukocyte influx during experimental glomerulonephritis	[68]
*Tek-Cre;Ndst1(f/f)*				Immuno	↓Ovalbumin-induced acute airway inflammation	[69]
*Tek-Cre;Ndst1(f/f)*				Immuno	↓Allergen-induced airway remodeling	[70]
*Tek-Cre;Ndst1(f/f)*				Immuno	↓Neutrophil trafficking	[71]
*Tek-Cre;Ndst1(f/f)*				Immuno	↓Acute renal allograft rejection	[72]
*Tie2-Cre;Ndst1(f/f)*				Immuno	→Neutrophil recruitment	[73]
*Tie2-Cre;Ndst1(f/f)*				Immuno	↓Th2 cytokines; ↓airway eosinophilia; ↓mucus secretion; ↓smooth muscle mass	[74]
*Alb-Cre;Ndst1(f/f)*					↑Triglyceride-rich lipoprotein particles	[75]
*Alb-Cre;Ndst1(f/f)*					↓Hepatic hepcidin expression; ↑iron accumulation in the liver and serum	[76]
*Alb-Cre;Ndst1(f/f)*					↑Accumulated plasma triglycerides	[77]
*Le-Cre;Ndst1(f/f),*					↓ERK signaling in lacrimal gland bud	[64]
*MMTV-Cre;Ndst1(f/f)*					↓Lobuloalveolar development in mammary gland	[78]
*Tie2-Cre;Ndst1(f/f)*					↓Pathogenesis of diabetic nephropathy	[79]
*Tie2-Cre;Ndst1(f/f)*					↓Diaphragm vascular development	[80]
*Wnt-1-Cre;Ndst1(f/f),*					↓ERK signaling in lacrimal gland bud	[64]
*Wnt1-Cre;Ndst1(f/f)*					↓Heart development	[63]
*Wnt1-Cre;Ndst1(f/f),*					→Lacrimal gland budding	[81]
*Alb-Cre;Ndst1(f/f);Apoe(−/−)*					↑Plasma triglyceride levels	[82]
*Krt14-rtTA/TC/RosaLSL/Ndst1(f/f)*					↓Corneal degeneration; ↓wound healing	[44]
*Ndst2(−/−)*			Neural	Immuno	↑Neurogenic inflammation	[83]
*Ndst2(−/−)*				Immuno	↓Histamine release upon IgE/anti-IgE challenge; ↓sulfated heparin; ↓connective-tissue type mast cells	[84]
*Ndst2(−/−)*				Immuno	↑Defective mast cells	[85]
*Ndst2(−/−)*					↑Tumor growth; ↑blood clotting	[86]
*Ndst2(−/−)*					↑Tumor growth	[87]
*Ndst2(−/−)*					↓Branching events in mammary gland	[88]
*Ndst2(−/−)*					↓Heparin-binding proteases; ↑plasminogen activation	[89]
*Ndst2(−/−)*					→Heart development	[63]
*Ndst2(−/−)*					→Heparin sulfate composition	[58]
*Ndst1(−/−);Ndst2(−/−)*				Immuno	↓Mast cell development	[85]
*Ndst1(−/−);Ndst2(−/−)*					↓Induction of adipocytes and neural cells; →osteoblast	[90]
*Ndst1(−/−);Ndst2(−/−)*					↓Endothelial cell development	[91]
*Ndst1(−/−);Ndst2(−/−)*				Immuno	↓Allergen-induced airway remodeling	[70]
*Ndst1(−/−);Ndst2(−/−)*				Immuno	↓Neutrophil trafficking	[71]
*MMTV-Cre;Ndst1(f/f);Ndst2(−/−),*					↓Abnormal branching events in mammary gland	[88]
*L7-Cre;Ndst1(f/f);Ndst2(−/−)*			Neural		↓Female reproductive behavior	[65]
*Wnt1-Cre;Ndst1(f/f);Ndst2(−/−)*					↓Heart development	[63]
*Le-Cre;Ndst1(f/f);Ndst2(−/−)*					↓ERK signaling in lacrimal gland bud	[64]
*Wnt1-Cre;Ndst1(f/f);Ndst2(−/−);*					↓Lacrimal gland budding	[81]
*Ndst1(−/−);Ndst3(−/−)*	Let				↓Embryonic development	[92]
*Pgr-Cre;Ndst1(f/f);Ndst2(−/−);Ndst3(−/−)*					Infertile	[93]
*Ndst4(−/−)*					↑Goblet cells; ↓colonocytes in the proximal colon; ↑apoptosis in the colonic epithelium	[94]
Glucuronic acid C5-epimerase						
*Glce(−/−)*		chondr			↓Development of kidney; ↓lung development; ↓skeletal development	[95]
*Glce(−/−)*		chondr			↑Hedgehog signaling in endochondral bones	[96]
*Glce(−/−)*				Immuno	↓Lymphoid organ development	[97]
*Glce(−/−)*				Immuno	↓B-cell maturation; ↓APRIL-mediated survival signals	[98]
*Glce(−/−)*					↓Heparin biosynthesis in mast cells	[99]
*Glce(−/−)*					↓Pericyte migration	[57]
*Glce(−/−)*					↓FGF2-induced proliferation in MEFs; ↓Erk phosphorylation	[100]
*Glce(−/−)*					↓Maturation of type I alveolar epithelial cells in embryonic lung; ↓vascularization in the developing lungs	[101]
HS 2-*O*-sulfotransferase						
*Hs2st(gt/gt)*	Let				Neonatal lethality; ↓Kidney development	[102]
*Hs2st(gt/gt)*					↓Development of metanephric mesenchyme	[103]
*Hs2st(−/−)*			Neural		↓Proper retinal ganglion cell-mediated axon formation	[104]
*Hs2st(−/−)*			Neural		↓Axon guidance	[105]
*Hs2st(−/−)*			Neural		↓Cerebral cortex; ↓Erk1/2 activation at the rostral telencephalic midline	[106]
*Hs2st(LacZ/LacZ)*			Neural		↓Migration of facial branchiomotor neurons in the hindbrain.	[107]
*Alb-Cre;Hs2st(f/f)*					↑Accumulated plasma triglycerides	[77]
*Emx1-Cre;HS2st(f/f)*			Neural		↓Translocation signals to astroglial precursors	[108]
*Zic4-Cre;HS2st(f/f)*			Neural		↓Translocation signals to astroglial precursors	[108]
*Tie2-Cre;Hs2st(f/f)*				Immuno	↑Binding to group B Streptococcus: ↓formation of neutrophil extracellular traps	[109]
*LysM-Cre;Hs2st(f/f)*				Immuno	→Neutrophil recruitment	[73]
*Tie2-Cre;Hs2st(f/f)*				Immuno	↑Th2 cytokines; ↑eosinophils	[74]
*Tie2-Cre;Hs2st(f/f)*				Immuno	↑Neutrophil recruitment	[73]
*Tie2-Cre;Hs2st(f/f)*				Immuno	↑Airway eosinophilia, ↑mucus secretion and smooth muscle mass in Alternaria-challenged allergic airway inflammation	[74]
*Le-Cre;Hs2st(f/f)*					↓Lacrimal gland development	[110]
*Le-Cre;Hs6st1(f/f);Hs6st2(−/−)*					↓↓Lacrimal gland development	[110]
*Le-Cre;Hs2st(f/f);Hs6st1(f/f);Hs6st2(−/−)*					↓↓↓Lacrimal gland development	[110]
HS 6-*O*-sulfotransferases 1–3						
*Hs6st1(−/−)*	Let				↓VEGF-A mRNA; ↓normal placentation; ↓skeletal development	
*Hs6st1(−/−)*			Neural		↓Retinal ganglion cell-mediated axon formation; ↑prolific inter-retinal innervation	[111]
*Hs6st1(−/−)*			Neural		↓Axon guidance	[105]
*Hs6st1(−/−)*			Neural		↑Erk activation; ↓corpus callosum development	[112]
*Hs6st1(−/−)*			Neural		↓Cranial axon patterning	[107]
*Hs6st1(−/−)*					↓Puberty maturation	[113]
*Alb-Cre;Hs6st1(f/f)*					↑Plasma triglycerides	[77]
*Alb-Cre;Hs6st1(f/f)*					↓Accumulated plasma triglycerides	[77]
*Hs6st2(−/−)*					↓Mast cell proteases in fetal skin-derived mast cells	[114]
*Hs6st2(−/−)*			Neural		↓Cranial axon patterning	[107]
*Hs6st1(−/−);Hs6st2(−/−)*					↓↓Mast cell proteases in fetal skin-derived mast cells	[114]
*Wnt1-Cre;Hs6st1(f/f);Hs6st2(−/−)*					→Lacrimal gland budding	[81]
HS 3-*O*-sulfotransferase 1						
*Hs3st1(−/−)*				Immuno	↑LPS-induced TNF-α sensitivity	[115]
*Hs3st1(−/−) on C57BL/6*	Let				↑Lethality	[116]
*Hs3st1(−/−) on C57BL/6/129 mix genetic background*					↓Anti-thrombin-binding sites in carotid artery; →tissue fibrin accumulation/coagulopathy	[116]
*Sulfatase 1/2*						
*Sulf1(−/−)*		chondr			↓Intervertebral disc homeostasis	[117]
*Sulf1(−/−)*			Neural		↑Motor neuron progenitor; ↓oligodendrocyte progenitor	[118]
*Sulf1(−/−)*			Neural		↓Cerebellum development; ↓neurite outgrowth deficits in neurons	[119]
*Sulf1(−/−)*			Neural		↓Conversion of motor neurons to oligodendrocyte precursor cells	[120]
*Sulf1(−/−)*			Neural		↑motor neuron progenitor; ↓oligodendrocyte progenitor	[120]
*Sulf1(−/−)*					→Kidney development; →skeletal development	[121]
*Sulf1(−/−)*					↓↓Corneal re-epithelialization	[122]
*Sulf1(−/−)*					↓Angiogenesis; ↑HS after myocardial infarction	[123]
*Sulf1(gt/gt)*					↑Accelerated ossification	[124]
*Sulf2(−/−)*			Neural		↓Brain development; ↓neuronal and behavioral plasticity	[125]
*Sulf2(−/−)*			Neural		↓Neurite outgrowth; ↑hydrocephalus	[125]
*Sulf2(−/−)*			Neural		↓Cerebellum development; ↓neurite outgrowth deficits in neurons	[119]
*Sulf2(−/−)*			Neural		↓Conversion of motor neurons to oligodendrocyte precursor cells	[120]
*Sulf2(−/−)*			Neural		↑Motor neuron progenitor; ↓oligodendrocyte progenitor	[120]
*Sulf2(−/−)*			Neural		↓Novel cell population (Olig2+Sox10-)	[126]
*Sulf2(−/−)*					↓Kidney development; ↓skeletal development	[121]
*Sulf2(−/−)*					↓Corneal re-epithelialization	[122]
*Sulf2(−/−)*					↓Angiogenesis; ↑HS after myocardial infarction	[123]
*Sulf2(gt/gt)*					↑Accelerated ossification	[124]
*Sulf2(gt/gt)*					↓Liver regeneration	[127]
*Olig2-Cre;sulf2(fl/fl);R26R-tomato*			Neural		↓Novel cell population (Olig2+Sox10-)	[126]
*Sulf1(−/−);Sulf2(−/−)*	Let				↓Kidney development; ↓skeletal development	[121]
*Sulf1(−/−);Sulf2(−/−)*		chondr			↑Accelerated ossification	[124]
*Sulf1(−/−);Sulf2(−/−)*			Neural		↓Axon guidance in the corticospinal tract.	[128]
*Sulf1(−/−);Sulf2(−/−)*			Neural		↓Motor function Sulf1/2 DKO mice on C57BL/6 and CD1d genetic background	[129]
*Sulf1(−/−);Sulf2(−/−)*					↑Glomerular cellularity; ↑albuminuria in streptozotocin-induced diabetic model	[130]
*Sulf1(−/−);Sulf2(−/−)*					↓Esophageal contractile	[131]
*Sulf1(−/−);Sulf2(−/−)*					↓Spermatogonial stem cells	[132]
*Sulf1(−/−);Sulf2(−/−)*					↓↓Corneal re-epithelialization	[122]
*Sulf1(gt/gt);Sulf2(gt/gt)*	Let	chondr			↓Bone development; ↓kidney development	[121]
*Sulf1(gt/gt);Sulf2(gt/gt)*		chondr			↓Body weight; ↑accelerated ossification	[124]
*Surfactant protein C-rtTA;TetO-Cre;Sulf1(f/f);Sulf2(f/f)*				Immuno	↑Neutrophil infiltration; ↑bleomycin-induced mortality	[133]

↓, Decrease; ↑, increase; →, no change; +, reported; f, flox; gt, gene trap; Let, lethal; chondr, Abnormal chondrogenesis; Neural, Neural disorders; Immuno, Immunomodulation.

## Data Availability

Not applicable.
